# Supplementary material for: Preventive and Curative Effects of Salicylic and Methyl Salicylic Acid Having Antifungal Potential against Monilinia laxa and the Development of Phenolic Response in Apple Peel
Source: Plants (Basel). 2023 Apr 8;12(8):1584. doi: 10.3390/plants12081584 (PMC10142601; doi:10.3390/plants12081584)
Supplement: Supplementary file 1 [file plants-12-01584-s001.zip › plants-2281999-supplementary.pdf]

SUPPLEMENTAL DATA

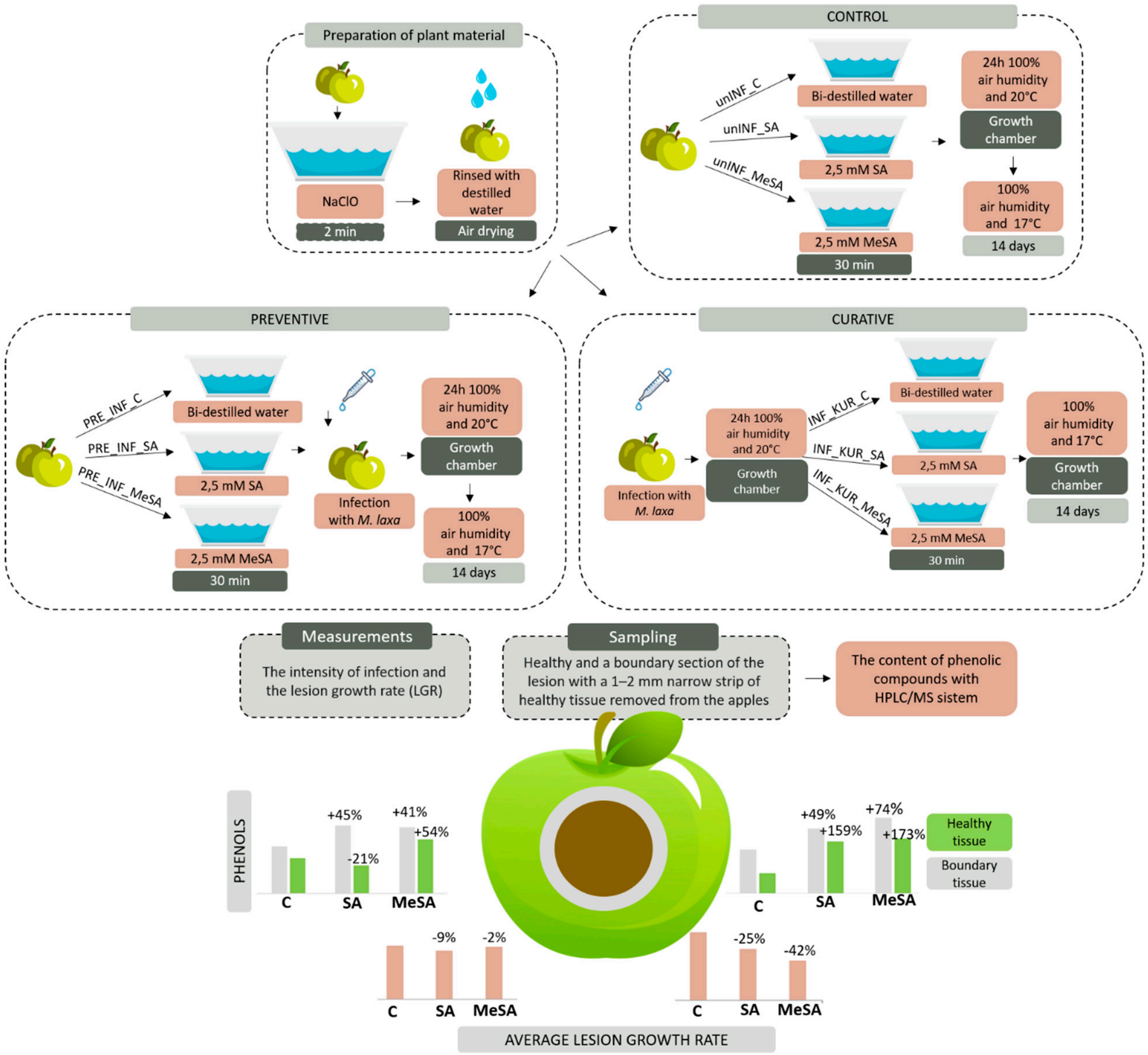

Figure S1: Graphical scheme of the experimental design with part of the presented results for total phenol content in healthy and boundary tissue around lesions caused by the fungus *Monilinia laxa*, treated preventively and curatively with salicylic acid (SA) and methyl salicylic acid (MeSA). The average lesion growth rate of *M. laxa* on preventively and curatively treated fruits with SA and MeSA is also presented.

Table S1: Content (mg/kg FW) of individual phenolic compounds and phenolic groups in apple peel infected with the fungus *Monilinia laxa* and treated with salicylic acid (SA) and methylsalicylic acid (MeSA).

Compaund	Tissue	Control			Preventive			Curative		
		**C**	**SA**	**MeSA**	**C**	**SA**	**MeSA**	**C**	**SA**	**MeSA**
Phloretin- 2-*O*-xylosyl glucoside	B				41.9±2.41 bc	56.05±3.48 bc	59.34±5.92 c	46.89±1.06 bc	60.03±5.27 c	61.11±2.71 c
	H	4.9±0.42 a	6.57±0.72 a	5.94±0.63 a	6.05±0.87 a	11.34±2.52 a	11.69±0.62 a	3.85±0.52 a	11.37±4.08 a	16.8±3.22 a
Phloridzin	B				29.74±1.24 cd	45.74±2.31 de	47.13±5.76 de	33.7±0.91 cd	48.83±5.26 e	49.37±2.7 e
	H	4.8±0.42 a	8.13±0.47 ab	5.73±0.47 a	6.2±1.18 a	9.087±1.69 ab	13.4±0.62 ab	3.19±0.45 a	14.4±4.75 ab	22.12±3.53 bc
3-hydroxyphloretin-2-xyloglucoside	B				9.69±0.66 ce	13.98±1.16 ef	12.47±1.44 def	8.73±0.47 bcd	15.89±1.8 fg	19.63±1.11 g
	H	4.19±0.5 ab	4.06±0.6 ab	3.59±0.62 a	3.4±0.43 a	5.09±0.8 ac	5.73±0.39 ac	4.04±0.33 ab	4.88±1.29 ac	6.66±1.55 ac
**Dihydrochalcones[1]**	**B**				**67±14.69 bc**	**115.8±6.1 de**	**118.9±12.1 de**	**89.31±1.54 cd**	**124.75±11.66 e**	**130.1±6.35 e**
	**H**	**13.9±0.33 a**	**18.76±1.3 a**	**15.26±1.32 a**	**15.65±2.6 a**	**25.55±3.63 a**	**30.82±0.97 a**	**11.1±0.87 a**	**30.66±8.29 a**	**45.61±7.46 ab**
p-coumaric acid hexoside derivative 1	B				0.47±0.09 ac	1.06±0.09 c	0.83±0.23 ac	0.64±0.08 ac	0.84±0.22 ac	1.04±0.08 c
	H	0.4±0.02 ac	0.6±0.1 ac	0.17±0.05 a	0.54±0.14 ac	0.47±0.1 ac	0.73±0.06 ac	0.3±0.02 ab	0.71±0.18 ac	1±0.31 bc
p-coumaric acid hexoside derivative 2	B				6.14±0.34 a	7.75±0.52 ab	8.23±0.33 ab	6.76±0.41 ab	6.88±0.79 ab	9.35±0.39 ac
	H	6.25±0.59 ab	8.29±1.12 ab	6.41±0.58 ab	6.78±0.74 ab	5.74±0.5 a	10.08±0.59 bc	5.88±0.58 a	8.51±0.51 ab	12.91±1.93 c
3-*p*-coumaroyl quinic acid	B				1.21±0.06 ade	1.68±0.14 cdf	1.77±0.13 df	1.44±0.18 bcde	1.7±0.37 cdf	2.42±0.1 f
	H	0.8±0.13 ac	0.52±0.23 ade	0.52±0.14 ab	0.86±0.13 ad	0.46±0.12 a	1.97±0.29 ef	0.86±0.09 ab	0.46±0.23 ade	2.01±0.26 ef
Neochlorogenic acid	B				11.91±0.82 ce	14.2±1.03 de	11.32±3.05 bce	10.32±1.05 ace	15.68±1.15 e	15.74±0.47 e
	H	4.96±0.93 ab	8.38±1.05 acd	8.8±1.47 acd	6.79±0.93 ac	9.26±0.92 ace	12.73±0.98 ce	4.67±0.59 a	11.91±1.24 ce	14.86±2.03 de
4-*p*-coumaroyl quinic acid	B				3.32±0.5 ab	8.53±0.83 ce	6.24±0.69 acd	4.65±0.65 ab	7.59±0.52 bce	8.89±0.29 de
	H	4.64±0.47 ab	4.65±0.69 acd	5.74±0.63 ac	4.89±0.57 ab	4.13±0.72 a	4.14±0.66 ce	4.45±0.47 a	8.18±0.69 ce	9.41±0.58 e
Chlorogenic acid	B				40.55±1.59 ef	53.28±2.06 fg	45.09±4.16 ef	34±0.88 de	48.71±3.67 fg	60.29±2.4 g
	H	4.04±0.45 a	13.14±1.96 ac	13.06±1.43 ac	13.31±2.21 ac	7.24±1.53 ab	19.92±1.2 bc	5.16±0.7 a	20.11±5.16 bc	22.3±4.86 cd
Cryptochlorogenic acid	B				6.8±0.78 acd	8.38±0.74 ce	10.61±1.2 e	7.6±0.73 ae	9.7±0.89 de	10.18±0.4 de
	H	4.55±0.51 ab	5.48±0.69 ac	4.028±0.49 a	4.65±0.6 ac	5.71±0.57 ac	8.44±0.42 ce	4.79±0.59 ac	7.95±1.06 bce	11.32±1.14 e
Caffeoyl synapoyl pentoside	B				0.8±0.09 acd	0.99±0.09 ce	1.25±0.14 e	0.9±0.09 ae	1.15±0.05 de	1.2±0.05 de
	H	0.54±0.06 ab	0.48±0.08 ac	0.65±0.06 a	0.55±0.07 ac	0.68±0.07 ac	1±0.05 ce	0.57±0.07 ac	0.94±0.12 bce	1.34±0.13 e

(to be continued)

(continuation of the Table S1)

		Control			Preventive			Curative		
		C	SA	MeSA	C	SA	MeSA	C	SA	MeSA
**Hydroxycinnamic acids[1]**	**B**				**71.21±5.33 def**	**95.86807±4.54 gh**	**85.33954±7.94 eg**	**65.79±4.48 cde**	**92.23437±6.66 fgh**	**109.10788±3.72 h**
	**H**	**26.18711±2.58 a**	**43.89936±3.57 abc**	**39.20112±3.43 ab**	**36.71±3.89 ab**	**33.68825±3.23 a**	**63.27836±3.23 cde**	**26.443±2.66 a**	**59.43123±8.04 bd**	**78.43±9.3 dg**
Q-3-rutinoside	B				4.28±0.49 ab	11.05±12.23 ad	6.84±0.35 abc	5.47±0.21 ab	5.14±0.26 ab	15.36±22.27 df
	H	4.98±0.42 ab	9.94±23.09 ad	12.31±12.25 bde	15.8±30.4 df	11.37±13.52 ad	19.41±13.26 ef	6.11±0.41 ab	22.71±23.56 cde	14.28±24.49 f
Q-3-galactoside	B				39.75±5.21 a	64.66±4.52 ab	51.39±3.52 a	34.93±2.39 a	37.15±1.34 a	78.3±7.94 abc
	H	42.51±2.73 a	66.61±7.99 abc	72.13±6.26 abc	113.1±22.91 cd	64.6±7.58 ab	133.41±16.23 d	44.34±3.28 a	130±8.03 d	98.91±12.34 bd
Q-3-glucoside	B				26.27±3.28 a	40.89±1.84 acd	34.98±2.26 ab	24.72±1.41 a	27.13±0.95 a	52.55±6.15 bcde
	H	23.63±1.44 a	39.52±5.12 ac	45.01±4.55 acd	60.07±9.51 cf	39.05±2.89 ac	76.73±6.02 f	25.35±1.83 a	73.73±4.95 ef	64.49±8.16 df
Q-3-xyloside	B				26.78±3.57 a	33.62±1.84 ab	31.41±2.16 ab	21.14±1.66 a	26.95±0.85 a	38.61±3.5 ab
	H	24.49±1.4 a	35.1±2.43 ab	36.93±4 ab	47.33±6.16 bc	29.66±2.46 a	65.56±6.42 d	23.88±1.71 a	63.67±3.2 cd	57.76±3.2 cd
Q-3-arabinopyranoside	B				4.25±0.6 ab	6.16±0.33 ac	5.15±0.35 ab	3.73±0.33 a	4.67±0.18 ab	7.11±0.72 bcd
	H	3.56±0.55 a	6.3±0.43 ac	7.04±0.78 bcd	8.52±1.09 ce	5.56±0.55 ac	11.44±1.13 e	3.83±0.28 a	11.18±0.46 e	9.84±0.46 de
Q-3-arabinofuranoside	B				81.8±11.66 ab	104.3±5.23 ac	97.8±6.93 ac	63.5±6.12 a	83.37±2.69 ab	116.86±10.83 bc
	H	77.2±4.45 ab	111.3±6.53 ac	112.1±12.64 ac	140.4±16.7 cd	88.4±8.62 ac	198.9±19.63 e	74.6±5.38 ab	196.6±16.26 e	178.5±7.35 de
Q-3-rhamnoside	B				100.1±12.2 ab	122.3±8.2 ac	113.8±7.86 ac	86.8±4.82 a	102.8±4.8 ab	140.9±12.17 ac
	H	99.48±5.05 ab	131.9±8.43 ac	151.9±16.39 bc	164.5±15.16 cd	113.7±8.9 ac	245.8±20.42 e	103.4±8 ab	252.5±14.9 e	216.7±13.24 de
**Flavonols[1]**	**B**				**283.1±36.88 ab**	**383±23.34 abc**	**341.3±23.07 ab**	**240.3±16.88 a**	**287.2±9.93 ab**	**449.7±43.5 bd**
	**H**	**275.8±15.36 ab**	**400.8±31.5 abc**	**437.4±45.41 bc**	**549.7±72.13 cd**	**352.3±30.76 ab**	**751.3±70.25 e**	**281.6±41.81 ab**	**750.4±41.81 e**	**640.5±41.81 de**
Epicatechin	B				148±13.4 c	203.2±70.58 de	207.4±19.47 de	163.8±10.26 cd	226.8±16.58 e	233.1±62.94 e
	H	21.8±3.08 a	30.93±4.44 ab	39.22±7.12 ab	29.26±4.56 ab	35.95±5.02 ab	56.01±2.2 ab	17.37±2.2 a	63.25±1.54 ab	77.1±1.76 b
Catechin	B				10.67±0.86 c	13.35±0.52 cd	11.3±1.04 c	10.04±0.58 c	12.2±0.92 cd	15.11±0.6 d
	H	1.01±0.11 a	3.29±0.49 ab	3.27±0.36 ab	3.34±0.56 ab	1.81±0.38 ab	5±0.3 b	1.29±0.176 a	5.04±1.29 b	5.42±1.33 b

(to be continued)

(continuation of the table S1)

		Control			Preventive			Curative		
		C	SA	MeSA	C	SA	MeSA	C	SA	MeSA
Procyanidin dimers[2]	B				379.2±38.28 cd	575.8±22.46 ef	568.5±56.37 ef	419±39.14 de	629±49.16 f	671±14.8 f
	H	86.5±13.03 a	113.1±14.61 ab	113.1±30.2 a	124.6±12.99 ab	130.8±14.9 ab	213.3±23.67 abc	90.3±9.54 a	231.1±40.51 abc	283.6±58 bd
Procyanidin trimers[2]	B				88.9±14.32 ae	133.8±9.94 def	147.9±17.39 ef	107.6±15.77 cdef	148.1±14.9 ef	168.4±5.2 f
	H	28.05±59.17 ab	44.59±9.15 abc	30.26±8.36 ab	35.71±5.64 ab	41.87±7.52 ab	90.91±7.25 be	25.18±4.74 a	78.53±16.44 abd	107.23±26.96 cdef
**Flavanols[1]**	**B**				**625.2±64.63 c**	**935.1±38.15 d**	**926.2±93.52 d**	**628.5±44.41 c**	**1016±80.8 d**	**1089±24.67 d**
	**H**	**137.3±21.59 a**	**225.5±25.42 a**	**180.4±49.21 a**	**185.4±23.12 a**	**210.5±26.61 a**	**365.2±26.61 ab**	**134.1±15.72 a**	**377.9±72.89 ac**	**522.4±84.65 bc**
**Total analyzed phenols[3]**	**B**				**1046±83.2 bc**	**1521±60.21 de**	**1481±125.6 de**	**1024±39.47 bc**	**1520±102.4 de**	**1777±66.69 e**
	**H**	**453.2±36.78 a**	**677.3±61.11 ab**	**588.6±150.4 ab**	**787.5±85.69 ab**	**622±51.95 a**	**1211±94.9 cd**	**470.7±48.5 a**	**1218.4±122.3 cd**	**1287±89.47 cd**

Abbreviations: C-control, SA-salicylic acid; MeSA-methyl salicylic acid; B-boundary tissue; H-healthy tissue; Q-quercetin

[1]Sum of all individual phenolic representatives of phenolic group, [2]Sum of all derivatives, [3]Sum of all identified phenolic compounds in apple peel

Data are means ± standard error. Different letters indicate significant difference between treatments ($p < 0.05$; Tukey test).
